# COPD and the Risk of Tuberculosis - A Population-Based Cohort Study

**DOI:** 10.1371/journal.pone.0010138

**Published:** 2010-04-13

**Authors:** Malin Inghammar, Anders Ekbom, Gunnar Engström, Bengt Ljungberg, Victoria Romanus, Claes-Göran Löfdahl, Arne Egesten

**Affiliations:** 1 Section for Infection Medicine, Department of Clinical Sciences Lund, Lund University, Lund University Hospital, Lund, Sweden; 2 Section for Respiratory Medicine, Department of Clinical Sciences Lund, Lund University, Lund University Hospital, Lund, Sweden; 3 Clinical Epidemiology Unit, Department of Medicine, Karolinska Institute and Hospital, Stockholm, Sweden; 4 Department of Epidemiology, Harvard School of Public Health, Boston, Massachusetts, United States of America; 5 Department of Clinical Sciences Malmo, Lund University, Malmo University Hospital, Malmo, Sweden; 6 Swedish Institute for Infectious Disease Control, Stockholm, Sweden; University of Cape Town, South Africa

## Abstract

**Background:**

Both chronic obstructive pulmonary disease (COPD) and tuberculosis (TB) primarily affect the lungs and are major causes of morbidity and mortality worldwide. COPD and TB have common risk factors such as smoking, low socioeconomic status and dysregulation of host defence functions. COPD is a prevalent co-morbid condition, especially in elderly with TB but in contrast to other diseases known to increase the risk of TB, relatively little is known about the specific relationship and impact from COPD on TB-incidence and mortality.

**Methods and Findings:**

All individuals ≥40 years of age, discharged with a diagnosis of COPD from Swedish hospitals 1987–2003 were identified in the Swedish Inpatient Register (n = 115,867). Records were linked to the Swedish Tuberculosis Register 1989–2007 and the relative risk of active TB in patients with COPD compared to control subjects randomly selected from the general population (matched for sex, year of birth and county of residence) was estimated using Cox regression. The analyses were stratified by year of birth, sex and county of residence and adjusted for immigration status, socioeconomic status (SES) and inpatient co-morbidities previously known to increase the risk of TB. COPD patients had a three-fold increased hazard ratio (HR) of developing active TB (HR 3.0 (95% confidence interval 2.4 to 4.0)) that was mainly dependent on an increased risk of pulmonary TB. In addition, logistic regression estimates showed that COPD patients who developed active TB had a two-fold increased risk of death from all causes within first year after the TB diagnosis compared to the general population control subjects with TB (OR 2.2, 95% confidence interval 1.2 to 4.1).

**Conclusions:**

This population-based study comprised of a large number of COPD patients shows that these patients have an increased risk of developing active TB compared to the general population. The results raise concerns that the increasing global burden of COPD will increase the incidence of active TB. The underlying contributory factors need to be disentangled in further studies.

## Introduction

Chronic obstructive pulmonary disease (COPD) and tuberculosis (TB) are both diseases that mainly affect the lungs and are major causes of morbidity and mortality worldwide.

One third of the world population is infected with *Mycobacterium tuberculosis* and eight million new cases of TB are reported every year [1]. In Sweden, as in many industrialised nations, the incidence of TB has declined over the past decades, presently being a low-incidence region with an annual incidence of around 5.5 cases/100,000 inhabitants, with a total of around 500 new cases annually. General vaccination against tuberculosis of newborns was performed from the 1940s until 1975 and thereafter selective vaccination has only been offered to children and young adults considered to run a high risk of exposure to TB. The majority of individuals who develop active TB in Sweden today are either relatively young immigrants from areas with a high incidence of TB or elderly native Swedes who were most probably infected at a younger age and suffer from reactivated TB [2].

The development of COPD results from a combination of polygenic inflammatory vulnerability and environmental factors, mainly tobacco smoke, leading to lung tissue remodelling and a non-reversible airflow limitation [3]. The prevalence of COPD is increasing worldwide, and it is estimated that COPD will become the third-leading cause of death by 2020[4]. In Sweden, approximately 500,000 individuals out of a population of 9 million suffer from COPD[5], and around 2,500 die from COPD annually [6].

TB and COPD have common risk factors such as smoking and low socioeconomic status [4], [7]. In addition, both diseases have a significant genetic vulnerability component, although little is known regarding the extent to which these traits are shared [8], [9]. In the present case-control study, 231,734 COPD patients and randomly-selected control subjects from the general population were compared to investigate whether an underlying diagnosis of COPD affects the risk of active TB and the impact of this co-morbidity on TB mortality.

## Methods

### Ethics

The study was approved by the Lund University Research Ethics Committee (590/2004 and 294/2007). Informed consent was not obtained since all information regarding individuals was made anonymous to investigators prior to analysis in accordance to Swedish regulations. [10].

### Setting

Swedish health care is publicly financed and all inpatient care is provided independently of health insurance and the patient's financial status. A unique, lifelong ten-digit personal identity number assigned to each person living in Sweden provides the possibility of linking records in databases administrated by the following federal institutes: Centre for Epidemiology (Swedish National Board of Health and Welfare), Statistics Sweden, and The Swedish Institute for Infectious Disease Control.

**The Swedish National Inpatient Register**, which was applied nationwide in 1987, includes individual information on all hospital discharges since 1964 with diagnosis coded according to The International Classification of Disease (ICD), Coding is performed by physicians at discharge and contra-signed by board-certified specialists. All records are controlled for general inadequacies and specific validation studies indicate that coverage is above 98% and that almost 90% of the diagnoses reported to the inpatient register are correct when compared with the original medical files[11].

**The Total Population Register** includes individual information on vital status, dates of immigration and emigration, county of residence, and country of birth.

**The Swedish National Population Censuses** performed in 1980, 1985 and 1990 provide information on housing conditions and socioeconomic status (SES). Participation was mandatory and response rates were 99.0%, 99.2% and 97.5%, respectively. Individuals were classified into different socioeconomic index groups (SEIs) based on type of occupation, educational background and responsibility levels according to specific criteria defined by Statistics Sweden. Subjects with missing information were mainly old age pensioners, unemployed, disability pensioners, homemakers and students[12].

**The Tuberculosis Register**: The Swedish Institute for Infectious Disease Control (SMI) monitors the epidemiology of infectious diseases in Sweden. All forms of TB have been notifiable according to law in Sweden since 1939. In 1969 a national tuberculosis register based on individual reports according to the communicable disease act was set up; since 1989 this register is administered by SMI. Reporting incident TB cases to the TB register is done in parallel by both the microbiological laboratories and clinicians when a patient is culture-positive. Clinically diagnosed TB is reported by clinicians only. The summarised statistics for the Tuberculosis Register during 1989–2007 is shown in TABLE 1.

**Table 1:** Summary statistics from Swedish Tuberculosis Register 1989–2007.

Tuberculosis in Sweden 1989–2007^1^	
No of TB cases	9,586 (100%)
Culture confirmed	7,893 (82%)
Pulmonary TB	6,234 (65%)
*Sex*	
Male	5,044 (53%)
Female	4,542 (47%)
*Immigration Status*	
Born in Sweden	3,761 (39%)
Born abroad	5,825 (61%)
*Age at TB-diagnosis*	
<40 years of age	4,310 (45%)
40–59 years of age	1,670 (17%)
60–79 years of age	2,367 (25%)
≥80 years of age	1,239 (13%)

1 Sources: The National Bacteriological Laboratory/Swedish Institute for Disease Control and Swedish Heart and Lung foundation. The Swedish Tuberculosis Index 1989–2002, ISSN 1103–4955. Tuberkulos I Sverige 2003–2008, in Swedish Only, in print.

### Population

The Centre for Epidemiology identified all individuals 40 years of age or older with a hospital discharge diagnosis of COPD between 1987–2003 in the Inpatient Register according to the International Classification of Diseases, Ninth and Tenth revisions (ICD-9: 491–492, 496; ICD-10: J41–J44), either as a main diagnosis (hospitalised *because of* COPD) or as secondary diagnosis (hospitalised *with* COPD). A total of 151,364 patients were identified, born 1880–1963, mean year of birth 1922. For each individual with a diagnosis of COPD, Statistics of Sweden randomly selected one control subject out of the general population from the Total Population Register matched for sex, year of birth, and county of residence during the year of first hospital discharge listing a COPD diagnosis.

### Follow Up

All patients and control subjects were linked to the Total Population Register to obtain information on country of birth, vital status and date of emigration. Each individual was also linked to The Swedish National Population Censuses of 1980, 1985 and 1990 to obtain information on SEI. The SEI groups were combined into three broad categories: manual, non-manual, and other types of occupation. Through linkage with the Inpatient Register we obtained information on all hospital discharges and all diagnoses between 1987 and 2003. To identify individuals with incident TB The Swedish Institute for Infectious Disease Control linked all individuals to the Tuberculosis Register from 1989 to 2007.

**Figure 1:**
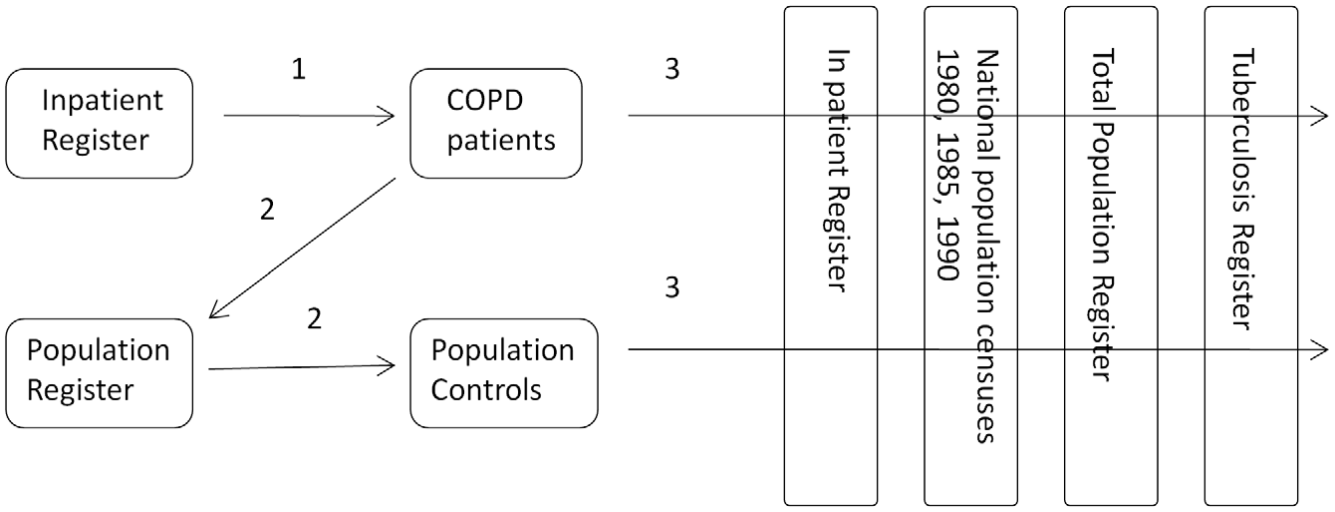
Flow chart demonstrating the study design. 1 = Identification of patients with COPD in the inpatient register, 2 = identification of their population controls (matched for age, sex and county of residence), 3 = Register linkage for vital status, inpatient co-morbidity, socio-economic position, prior and incident TB.

The primary outcome was defined as the first episode of any form of TB notified in the Tuberculosis Register in the overall analysis of TB, and the first episode of TB per localisation (pulmonary and extra-pulmonary) in the analysis by localisation. Individuals with more than one episode were counted only once. Follow up started one year after the first hospitalisation with COPD and on the corresponding date for control subjects to account for the fact that early TB symptoms may mimic COPD. Follow up ended with the first episode of TB, date of emigration, date of death or 31 December, 2007, whichever came first. A flow chart showing the principles of the study design is shown in Figure 1.

### Exclusion

Among the 151,364 COPD patients, 101 (0.07%) individuals were excluded because of data-irregularities. Another 27,357 (18.1%) COPD patients, who died during the first year after COPD diagnosis, were excluded since they had a follow-up time of less than a year. 749 (0.49%) COPD patients were excluded from the analysis because of TB or TB-sequelae prior to or during their first year of hospitalisation with COPD identified in either the Tuberculosis Register (n = 212) or the Inpatient Register (n = 537) 1987–2003 (ICD-9: 010–019, 131; ICD-10: A15–A19, B90). 7,216 (4.8%) control subjects had a follow up time of less than a year, 162 (0.11%) control subjects were excluded because of TB or TB-sequelae prior to inclusion identified in either the Tuberculosis Register (n = 68) or the Inpatient Register (n = 94) 1987–2003.

As controls were randomly selected from the background population, some of the COPD patients also appeared as control subjects with time. We excluded 2,528 (1.6%) who had been hospitalised with a COPD-diagnosis prior to inclusion. Another 3,935 (2.7%) control subjects were initially included in the analysis but censored by the date of the first hospitalisation listing COPD. Because of the matched design 7,290 (4.8%) COPD-patients lacking controls due to exclusions and 25,591 (16.9%) odd controls were excluded. Analysis was based on the remaining 115,867 case-control pairs. Baseline characteristics of the study populations are shown in TABLE 2.

**Table 2:** Demographic characteristics of the study populations.

	COPD; n = 115,867 (100%)	Controls; n = 115,867 (100%)
*COPD Classification*		
Primary diagnosis	70,404 (60.8%)	
Additional diagnosis	45,463 (39.2%)	
*Sex*		
Male	62,226 (53.7%)	
Female	53,641 (46.3%)	
*Year of birth*		
1888–1919	44,560 (38,5%)	
1920–1939	59,821 (51,6%)	
1940–1963	11,486 (9,9%)	
*Age Distribution^1^*		
(at COPD diagnosis)		
40–59 years of age	15,756 (13.6%)	
60–79 years of age	72,658 (62,7%)	
≥80 years of age	27,453 (23.7%)	
*Immigration status*		
Born in Sweden	103,045 (88.9%)	105,950 (91.4%)
Born abroad	12,822 (11.1%)	9,916 (8.6%)
*Socioeconomic Status*		
Non-manual	21,141 (18.3%)	30,092 (26.0%)
Manual	33,197 (28.6%)	31,385 (27.1%)
Other	11,569 (10.0%)	12,695 (11.0%)
Outside workforce	49,960 (43.1%)	41,695 (35.9%)

1 Control subjects were matched for year of birth, sex and county of living during the year of first hospital discharge listing COPD.

### Statistical analysis

Cox proportional hazards models were used to estimate hazard ratios (HR) of subsequent TB, comparing patients with COPD and control subjects. HRs were calculated separately for pulmonary, extra-pulmonary and pooled TB infections. All models were internally stratified by year of birth, sex and county of residence and subsequently adjusted for SES, co-morbidity and immigration status, dichotomised by birth in Sweden (yes/no). Co-morbidity was defined as prior hospital discharge diagnosis of diabetes mellitus, cardiac disease, alcohol liver disease or alcohol abuse, or silicosis, according to ICD-9 or ICD-10, as well as total number of hospitalizations and total duration of hospital stay. Interactions between COPD-status and age at inclusion, year of birth, sex, SES, immigration status, the level of diagnosis and prior hospitalization on outcome were tested by entering interaction terms to the fully adjusted COX model. Baseline predictors of TB among COPD patients were estimated using Cox regression stratified by year of birth among those born after 1915 who were not old age pensioners when the population censuses were performed. In the sub-population of patients with TB, logistic regression adjusted for sex and age at TB-diagnosis was used to estimate the OR of one-year all-cause mortality between COPD patients and control subjects. Proportionalities of hazards were assessed graphically and by testing for a non-zero slope in a generalised linear regression of the scaled Schoenfeld residuals as a function of time. All analyses were performed using STATA/SE (version 10.1 for Windows; StataCorp LP, College Station, TX).

## Results

### Relative risk of TB in COPD patients versus the general Population

Among the 231,734 cases and controls, a total of 291 first episodes of TB occurred - 201 among COPD patients and 90 among the control subjects during 680,818 and 986,119 person-years of follow-up, respectively, as detailed in TABLES 3 and 4. The resulting crude incidence rates of TB were 3.0 per 10,000 person-years of follow-up (95% confidence interval [95% CI] 2.6 to 3.4) in COPD patients and 0.9 (95% CI 0.7 to 1.1) in control subjects. In absolute terms this corresponds to one excess TB case among COPD patients per 4,900 person-years. The age-, sex- and county-stratified hazard ratio (HR) estimated by Cox regression was 3.3 (95% CI 2.5 to 4.2). Adjustment for immigration status, SES and co-morbidity resulted in a HR ratio of 3.1 (95% CI 2.4 to 4.1); see TABLE 5 for details.

**Table 3:** In-patient care and co-morbidity at inclusion.

	COPD; n = 115,867 (100%) (n = 115,867; 100%)	Controls; n = 115,867 (100%)
Number of hospital discharges		
Mean/median	3.8/2	1.8/1
Number of days spent in hospital		
Mean/median	32.4/8	19.1/1
*Co-morbidity*		
Alcohol consumption^1^	4,671 (4.0%)	1,070 (0.9%)
Cardiac disease^2^	25,509 (22.0%)	12,463 (10.8%)
Diabetes mellitus^3^	6,814 (5.9%)	4,092 (3.5%)
HIV	119 (0.10%)	9 (0.01%)
Silicosis	44 (0.04%)	9 (0.01%)
Renal failure	730 (0.6%)	259 (0.2%)

1 Including alcohol abuse and/or alcohol-induced liver disease.

2 Including ischemic heart disease and cardiac failure.

3 Including diabetes mellitus type 1 and 2.

**Table 4:** Characteristics of patients suffering from tuberculosis in the presence and absence of COPD as a pre-morbid condition.

	COPD	Controls
No of TB cases	201 (100%)	90 (100%)
Positive culture	158 (78.6%)	79 (87.8%)
*Localization*		
Pulmonary	157 (78.1%)	51 (56.7%)
Extra-pulmonary	26 (12.9%)	28 (31.1%)
Both	18 (9.0%)	11 (12.2%)
*Sex*		
Male	127 (63.2%)	59 (65.6%)
Female	74 (36.8%)	31 (34.4%)
*Immigration status*		
Born in Sweden	149	74
Born abroad	52	16
Age at TB-diagnosis in years		
mean/median	75.2/77	77.8/78
40–59	14	5
60–79	117	47
≥80	70	38
No of deaths	93 (46.2%)	29 (32.2%)
(within first calendar year after diagnosis)		
*Age at death*		
*(within first calendar year after diagnosis)*		
mean/median	77.8/79	81.9/82
40–59	4	0
60–79	46	10
≥80	43	19

**Table 5:** Hazard ratios for TB among COPD-patients (n = 115,867) compared to general population control subjects (n = 115,867).^1^

	Univariate HR (95%CI)	Multivariate HR (95%CI)^2^
Control subjects	1	1
COPD-patients	3.26 (2.53–4.20)	3.14 (2.42–4.08)
*Socioeconomic Status*		
Non-manual occupation	1	1
Manual occupation	0.99 (0.68–1.44)	0.86 (0.59–1.24)
Other occupation	1.01 (0.63–1.63)	0.98 (0.61–1.58)
Missing information	2.04 (1.40–2.94)	1.40 (0.97–2.04)
*Immigration Status*		
Born in Sweden	1	1
Born abroad	3.55 (2.65–4.74)	3.08 (2.29–4.15)
*Co-morbidity*		
Alcohol over-consumptionconsumption^3^	1.38 (0.66–2.88)	1.06 (0.50–2.26)
Cardiac disease^4^	1.00 (0.71–1.42)	0.85 (0.57–1.26)
Diabetes mellitus^5^	1.13 (0.62–2.09)	1.12 (0.60–2.10)
*Inpatient care* 6		
No of days spent in hospital		
0	1	1
1–29	1.04 (0.80–1.34)	1.41 (0.19–10.38)
≥30	1.09 (0.76–1.55)	1.62 (0.21–12.36)
No of hospital discharges		
0	1	1
1–4	1.02 (0.79–1.32)	0.61(0.08–4.46)
≥5	1.18 (0.84–1.67)	0.52 (0.07–4.06)

1 Estimated by COX regression stratified for year of birth, sex and county of residence, using calendar time as time scale.

2 Adjusted for all parameters in the table.

3 Including alcohol abuse and alcohol-induced liver disease.

4 Including ischemic heart disease and cardiac failure.

5 Including diabetes mellitus type 1 and type 2.

6 Prior to inclusion.

There was no evidence of effect modification by age at inclusion (p = 0.26), year of birth (p = 0.89) sex (p = 0.50), SES (p = 0.19), immigration status (p = 0.77) or the level of diagnosis (i.e. whether the COPD diagnosis was primary or secondary) (p = 0.13). A stratified analysis, restricted to those pairs of COPD-patients/controls where the control subject had been hospitalized prior to inclusion (n = 118,570), thus using an in-patient control group yielded similar results, with an HR of 3.06 (95% CI 1.98–4.73). A subgroup analysis between this subgroup and the subgroup of pairs where the control had no prior hospitalization showed no significant difference between the groups, (p = 0.93 from likelihood ratio test for interaction). A sensitivity analysis was performed wherein control subjects who were later hospitalised for COPD were not censored, thus analysing them as both COPD patients and controls. This analysis yielded a similar result after stratification for age, sex, and county of residence, adjusted for co-morbidity, SES and immigration status, resulting in a HR of 3.0 (95% CI 2.3–3.9). An additional analysis was performed in the 35,497 excluded case-control pairs, in whom 412 TB cases occurred during 1989–2003 (310 in COPD-patients and 102 in controls). Conditional logistic regression yielded OR 2.9 (2.3–3.7) adjusted for immigration status.

### Localisation of TB: Comparing COPD patients and Controls

Pulmonary TB was seen in 175 COPD patients and 64 control subjects, corresponding to an HR of 3.7 (95% CI 2.9 to 5.1) when stratified by age, sex and county and adjusted for immigration status, co-morbidity and SES. Extra-pulmonary TB was seen in 44 COPD patients and 39 control subjects resulting in an HR of 1.7 (95% CI 1.0 to 2.6) stratified by age, sex and county and fully adjusted.

### Mortality after TB within first year

Of the 291 patients who developed TB, 122 died within the first calendar year after the TB-diagnosis; 93 of the 201 COPD patients and 29 of the 90 control subjects. Median age at death was 79 years of age among COPD patients and 82 years of age among control subjects. COPD patients with TB had an odds ratio (OR) with respect to death of 2.2 (95% CI 1.3 to 3.9) adjusted for age at TB-diagnosis, compared to general population controls with TB. Cardiac disease and SES were the only covariates significantly associated with death in univariate analysis and none of the investigated co-morbidities, immigration status, sex nor SES covariates reached significance in multivariate analysis (data not shown). Estimation of the OR, with respect to death within two years after TB-diagnosis, yielded similar results.

### Predictors of TB among COPD patients

217 TB cases occurred among the 86,920 COPD patients born after 1915 who were not old age pensioners when the population censuses were performed. Hence missing information on SES was equal to being outside the workforce mainly as disability pensioner, unemployed, homemaker or student. Immigration status, sex and SES, all predicted the risk of TB in univariate analysis stratified by year of birth. In the multivariate model stratified for year of birth and adjusted for all of the above parameters, immigration status, sex and SES remained significant; see TABLE 6 for details. The younger birth cohorts had a lower absolute incidence of active TB compared to the older birth cohorts but there was no effect modification by birth cohort.

**Table 6:** Predictors of TB among individuals discharged with a prior diagnosis of COPD (n = 86,920).^1^

	Univariate HR (95% CI)	Multivariate HR (95% CI)^2^
*Immigration status*		
Born in Sweden	1	1
Born abroad	3.19 (2.25–4.53)	3.11 (2.18–4.44)
*Sex*		
Male	1	1
Female	0.72 (0.52–1.00)	0.66 (0.47–0.91)
*Socioeconomic Status*		
Non-manual occupation	1	1
Manual occupation	0.71 (0.46–1.10)	0.64 (0.42–0.99)
Other	0.65 (0.35–1.22)	0.61 (0.32–1.14)
Outside workforce	1.24 (0.81–1.89)	1.10 (0.72–1.68)
*Co-morbidity*		
Alcohol over-Consumption^3^	1.08 (0.47–2.47)	0.96 (0.42–2.22)
Cardiac disease^4^	0.91 (0.58–1.41)	0.86 (0.54–1.35)
Diabetes mellitus^5^	0.87 (0.38–1.97)	0.85 (0.37–1.96)
*Age at inclusion*		
*(first hospitalisation listing COPD)*		
40–59	1	1
60–79	1.58 (0.59–4.21)	1.57 (0.59–4.18)
≥80	1.06 (0.28–3.95)	1.08 (0.29–4.05)

1 Estimated by Cox regression, stratified for year of birth, using calendar time as time scale.

2 Adjusted for all parameters in the table.

3 Including alcohol abuse and alcohol-induced liver disease.

4 Including ischemic heart disease and cardiac failure.

5 Including diabetes mellitus type 1 and 2.

## Discussion

Previous studies have shown that COPD is a frequent co-morbid condition in TB patients [13], [14], [15]. However, to the best of our knowledge there is no study that has specifically studied the association between COPD and subsequent TB. In this population-based study comprised of 115,000 COPD patients, we show that individuals with a hospital discharge diagnosis of COPD have a three-fold increased risk of developing active TB compared to the general population, mainly due to an excess risk of pulmonary TB. Moreover, our study shows that TB-patients with COPD have a two-fold increased risk of death within first year after TB-diagnosis when compared to general population control subjects suffering from TB.

The national, publicly-financed health care system ensures that the present study covers all patients hospitalised with COPD in Sweden during the observation period. However, COPD patients who were only treated as outpatients were not included in the analysis. The prevalence of physician-diagnosed COPD in Sweden is estimated to be around 5% [16], [17], [18]. Of the randomly selected general population control subjects, 4.3% did have a hospital discharge diagnosis of COPD during the observation period which implies that a large proportion of patients with COPD will eventually be hospitalised.

Validation of the COPD diagnosis was not possible due to anonymisation of data prior to analysis, but there was no evidence for an effect modification by the level of diagnosis, suggesting that the diagnosis of COPD, whether primary or secondary, was equally accurate. Swedish validation studies of hospital discharge coding suggest a slight overlap between COPD and asthma [19]. Previous studies have associated a history of asthma with a decreased risk of TB [20], [21], [22], [23]. In the present study, a misclassification of the COPD-diagnosis, in addition to the inclusion of COPD patients receiving only outpatient care as control subjects, would presumably bias the estimates downwards.

A limitation of the present study is the inability to estimate the risk of exposure to TB, previous or current. In addition, it was not possible to identify individuals who suffered from latent tuberculosis infection or who were recently exposed to TB at the time of the study entry. Individuals identified as having had TB or TB-sequelae were excluded from analysis since pulmonary TB itself can lead to COPD [24]. Comparison of the Inpatient register and the TB register for the overlapping time periods (1989–2003) yielded around 70% excess TB-diagnoses in the Inpatient register. The TB register contains valid incident cases whereas the Inpatient register probably also contains cases of former TB and cases examined for TB but, but this incongruence needs to be further evaluated. We used the TB register as the golden standard of incident TB but we still used a diagnosis of TB in the Inpatient Register prior to inclusion as an exclusion criterion to be certain that former TB cases were properly excluded.

Models were adjusted for the use of inpatient care at baseline and inpatient co-morbid conditions associated with COPD and previously known to increase the risk of active TB [25], [26]. Residual confounding from alcohol intake or co-morbid conditions only requiring outpatient care cannot be ruled out. HIV, renal failure or silicosis were not included in the models since these were not listed in the hospital discharge diagnoses of TB-cases. Globally, HIV infection is an important modifier of the risk of active TB but the impact in the Swedish setting is probably only of minor importance since the prevalence of HIV-infection is low (around 0.05%) [27].

There is growing consensus that smoking increases the risk active TB-disease[28]; three different meta-analyses essentially covering the same studies found a 1.5–2 fold increased risk of TB among current smokers [29], [30], [31]. A limitation of the present study is the inability to control for smoking, which was not possible since none of the registers with national coverage contain smoking data. In this study, although on a group level, COPD-patients with an ever-smoking rate of around 75% [32], [33] are compared with general population control subjects with an ever smoking rate of around 50% [34].

Smoking prevalence in Sweden as reflected in the control group has declined during the past decades[34]. Smoking rates have gone from 35% and 28% in men and women in 1980 to 14% and 18% in 2004, respectively. The birth cohorts of the 1940s and 1950s have the highest smoking prevalence and smoking habits have been shown to be strongly associated with SES and immigration status[34]. Prevalence studies of COPD in Sweden have found that current smoking rates range between 31% to 47% of people with physician-diagnosed COPD (not necessarily hospitalised) [32], [33], whereas in a study of patients hospitalised for severe COPD (GOLD-stage IV) only 9% were current smokers [35]. The smoking rate by the first hospitalisation for COPD has not been studied.

Despite the inability to control for smoking, our results are in line with previous studies. A case-control study of from England [36] found that patients with emphysema and bronchitis had a two to three-fold increased risk of developing TB, although the results were based on a small sub-group of individuals suffering from emphysema and bronchitis (n = 264). A cohort-study from Denmark with 14,000 participants [37] also found a two to three-fold increased RR for hospitalisation with TB for participants with moderate to severe lung function impairment compared to participants with normal lung function.

Apart from smoking, COPD patients suffer from conditions that could potentially increase the risk of active TB such as low body mass index [38] and impaired muco-ciliary clearance. Several bacterial species are important both as colonisers and as inducers of COPD exacerbations [39]. Factors promoting the survival of these bacteria in the lower airways are likely to promote the survival of *M Tuberculosis* as well. In addition, patients suffering from COPD-exacerbations are often treated with oral corticosteroids, a well known risk factor for TB [36].

There are some conflicting findings in the literature as to whether COPD is a risk factor for death from TB [40], [41]. We found a two-fold increased risk of all cause mortality for COPD patients within first year after TB the diagnosis. This could be due to death from other causes related to COPD, caused by worsened outcome because of delayed diagnosis or speculatively, altered host defence functions in the lower airways.

This population-based study, comprising a large number of COPD patients, shows that COPD patients have an increased risk of developing active TB compared to the general population. These results raise concern that the increasing global burden of COPD will further enhance the incidence of active TB especially in settings with a high burden of latent TB. The underlying contributory factors such as smoking, low body mass index, impaired muco-ciliary clearance, treatment with corticosteroids or impaired host defense functions need to be disentangled and evaluated in further studies.
